# Ideal Configuration Distribution of Femoral Neck Cannulated Screw in High Altitude Population: A Finite Element Study

**DOI:** 10.1155/2022/2085378

**Published:** 2022-10-26

**Authors:** Kai Lei, Dechun Li, Rong Ren, Jingtang Bai, Yuanyuan Qi, Xuebin Zhang

**Affiliations:** ^1^Department of Traumatic Orthopedics, Qinghai University Affiliated Hospital, China; ^2^Department of Joint Surgery Department, Qinghai University Affiliated Hospital, China; ^3^Department of Traumatic Orthopedics, The Third Hospital of Hebei Medical University, China

## Abstract

**Objective:**

For people in high altitude areas, the shape of the femoral neck is different due to the different living environment. To deduce the optimal insertion angle and position of the cannulated screw using three-dimensional finite element technology in patients at high altitude.

**Methods:**

The data of 100 volunteers were used to establish a finite element model. The stress and displacement of cannulated screws of equilateral and oblique triangle screw placement in femoral neck fracture model and complete femoral neck model were evaluated.

**Results:**

On the narrowest plane, the average values of ∠A, B, and C were 47.63^o^, 75.49^o^, and 56.88^o^, respectively, and the shape was an oblique triangle. In complete femoral neck model, the maximum Von Mises stress of the three cannulated screws of equilateral triangle screw placement was slightly larger than oblique triangle screw placement, and this difference was more obvious in femoral neck fracture model. Under same loads, the overall maximum displacement of femur in oblique triangle screw placement was less than equilateral triangle screw placement in two models.

**Conclusions:**

For people in high-altitude areas, the three screws should be implanted in an oblique triangle configuration in cannulated screw treatment of femoral neck fractures.

## 1. Introduction

Femoral neck fractures are common clinical fractures, accounting for 3.6% of total body fractures and 50% of hip fractures [1]. It is estimated that by 2050, the number of femoral neck fractures in the world will exceed 63 million [2], which will cause a huge burden on society, families, and hospitals. The annual cost of treating femoral neck fractures in the United States exceeds $12 billion [3]. The vast majority of patients with femoral neck fractures require surgical treatment. If the femoral neck fracture is not properly treated, it will often lead to serious consequences due to early internal fixation failure, avascular necrosis of the femoral head, nonunion, and other complications, which will bring serious economic losses to the society and family [4]. Currently, hip arthroplasty is generally recommended for patients with displaced femoral neck fractures over 65 years of age and internal fixation is recommended for patients younger than 65 years of age. Among various internal fixation methods, percutaneous cannulated screw fixation is most widely used [5].

At present, with the in-depth research on femoral neck fractures, there are many ways to place cannulated screws in the treatment of femoral neck fractures; however the most ideal configuration has not been determined yet. The configuration of the cannulated screw should conform to the normal physiological shape of the femoral neck to obtain better stability.

For people in high altitude areas in Qinghai Province (between 1500-3500 meters above sea level), especially the Tibetan people, the shape of the femoral neck are different due to the different living environment and living habits. The purpose of this study was to use three-dimensional finite element technology to evaluate the morphology of the proximal femur in patients at high altitude, and to deduce the optimal insertion angle and position of the screw, which provides a scientific basis for the treatment of clinical femoral neck fractures in high altitude areas.

## 2. Materials and Methods

Volunteers who met the standards in Qinghai high-altitude areas were included. The inclusion criteria were volunteers who were 18-65 years old with long-term living in high altitude areas (1500-3500 meters). The exclusion criteria were (1) patients with old or fresh fracture of the femoral neck, rheumatism, congenital hip dysplasia, bone tumors, and other bone diseases; (2) pregnant and lactating women; and (3) patients with lower limb dysfunction.

100 volunteers were screened out, of which 28 were male, with 64 cases of left femoral neck and 36 cases of right femoral neck, aged 38-65 years old. This study was approved by the hospital. All participants provided written informed consent.

### 2.1. Finite Element Model

**Figure 1:**
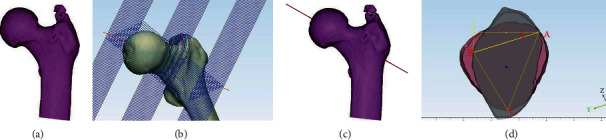
(a) The threshold growth command (region growing) was used to separate the femur, and then the three-dimensional model of the separated femur was reconstructed to obtain the three-dimensional model. (b) This line is the central axis of the femoral neck. (c) The conversion command (translate) was used to generate multiple parallel planes in the femoral neck section with a 2 mm interval as the cutting surface, and the cut command (cut) was used to cut the femoral neck with a thickness of 2 mm. (d) The intersection of the two plane boundaries is taken as point A on the anterior upper side, point B is a point on the posterior upper side, which is higher than the rearmost cortex, and point C is the lowermost point, which is close to the lowermost cortex. Use these three points to make a triangle, and cross points A and B to make a horizontal line and a vertical line, respectively. The angle between line AB and the horizontal line was Angle a.

The DICOM format files of volunteers obtained by continuously scanning with a 256-slice spiral computed tomography (CT) were imported into the Mimics Research 21 software. Using the new mask function, a mask was created according to the gray value difference of different structure organizations. Using the edit masks tool, the redundant part of the femoral head and the acetabulum was cut off and the entire femur was edited. The threshold growth command (region growing) was used to separate the femur, and then the three-dimensional model of the separated femur was reconstructed to obtain the three-dimensional model (Figure 1(a)).

### 2.2. Cutting

A contour line of the model was generated using the polylines command, and a sphere according to the contour line of the femoral head was then generated. Take the center of the sphere and mark it as point A, and then take the center line (Fit Centerline), and mark the left side of the intersection of the two centerlines as point B. A straight line (draw line) was drawn through the point A and B. This line is the central axis of the femoral neck (Figure 1(b)). This file was saved and then imported to 3-matic Research 13.0, and the plane perpendicular to the center line of the femoral neck (DatumPlane-001) was made using the Create Datum command (Create Datum Plane). The conversion command (translate) was used to generate multiple parallel planes in the femoral neck section with a 2 mm interval as the cutting surface (Figure 1(c)), and the cut command (cut) was used to cut the femoral neck with a thickness of 2 mm.

### 2.3. Data Sharing Policy

The narrowest plane of the femoral neck in the horizontal and coronal positions was found, respectively. The other planes were hidden; the two narrowest planes were placed in the vertical direction along the central axis; and adjust the transparency of one layer to middle (Figure 1(d)). The file was export in screen capture format. Digimizer Version 4.2.6.0 was used to select the three-point position of the cannulated screw and measure the Angle a. Import the screenshot file and select three points and the three-point selection criteria are as follows: the intersection of the two plane boundaries is taken as point A on the anterior upper side, point B is a point on the posterior upper side, which is higher than the rearmost cortex, and point C is the lowermost point, which is close to the lowermost cortex. Use these three points to make a triangle, and cross points A and B to make a horizontal line and a vertical line, respectively. The Angle between line AB and the horizontal line was Angle a, and the average value of Angle a in 100 cases was statistically analyzed.

### 2.4. Finite Element Analysis

With 3-matic Research 13.0, the angle between the fracture line and the horizontal was made to 70°to design a Pauwels type III femoral neck fracture model. 3-Matic Research 13.0 was used to design the femoral neck fracture model, which was the complete model. For the femoral neck fracture model, the plane perpendicular to the axis of the femoral neck was taken as the cutting plane to perform a complete cut in the middle part of the femoral neck to simulate the femoral neck fracture (Figure 1(c)).

UnigraphicsNX8.5 (Siemens PLM software) software was used to reconstructs the cannulated lag screw model with diameter of 6.5 mm and thread length of 16 mm. 3-matic Research 13.0 was then used to implant three cannulated screws into the femoral neck fracture model for fracture fixation. We adopted the oblique triangle and equilateral triangle screw placement methods to simulate the screw fixation. The angle of the oblique triangle was determined according to the measurement results in Figure 1(d).

The bone is considered to be uniform and has isotropic linear elastic properties in the finite element analysis using the Abaqus software [6–9]. The values of Poisson's ratio and elastic modulus were shown in Table 1. The cannulated screw is made of Ti-6AL-4 V titanium alloy, and the finite element model was meshed with tetrahedral 10-node elements. Throughout the simulation process, the influence of gravity is considered to be negligible. There was friction between bone and screw and between bone pieces. The threaded surface of the cannulated screw was considered to be the connection constraint. The friction coefficient between the bone and the screw was taken as 0.3, and the friction coefficient between the bone pieces was taken as 0.46 [10], and the distal end of the femoral surface was restricted by zero degrees of freedom. Loads of 500 N, 1000 N, 1500, and 2100 N were applied to the finite element model, with the maximum equivalent to 300% of the body weight, to simulate postoperative gradual weight bearing. The Von Mises stress and displacement changes of the cannulated screw and the stress distribution of the femoral head were recorded.

## 3. Results

### 3.1. Measurement Results

The detailed measurement results were shown in Table 2. On the narrowest plane, the average values of ∠A, B, and C were 47.63^o^, 75.49^o^, and 56.88^o^, respectively, and the average value of ∠a was 20.26, and the shape was an oblique triangle. However, the minimum and maximum values of ∠a were 15.43 and 24.67, respectively, which are not much different, and the standard deviation is 2.33.

**Figure 2:**
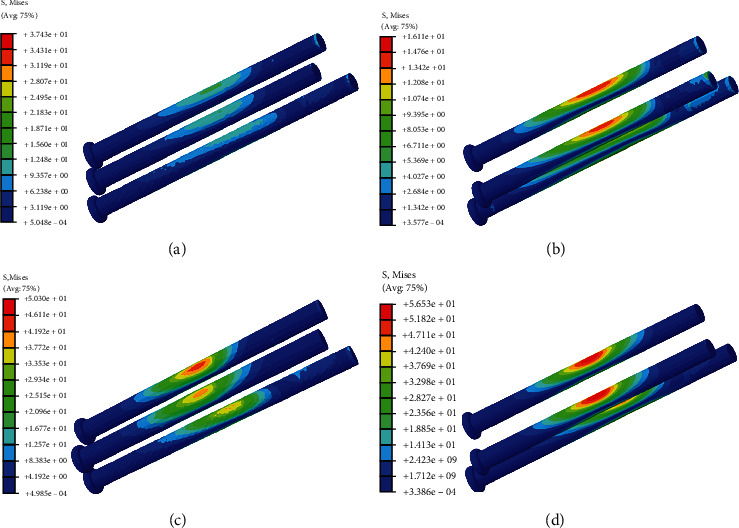
The stress nephogram of cannulated screws under loads of 500 N in two models. Red is the maximum stress. (a). Oblique triangle screw placement in complete femoral neck model. (b). Equilateral triangle screw placement in complete femoral neck model. (c). Oblique triangle screw placement in femoral neck fracture model. (d). Equilateral triangle screw placement in femoral neck fracture model.

**Figure 3:**
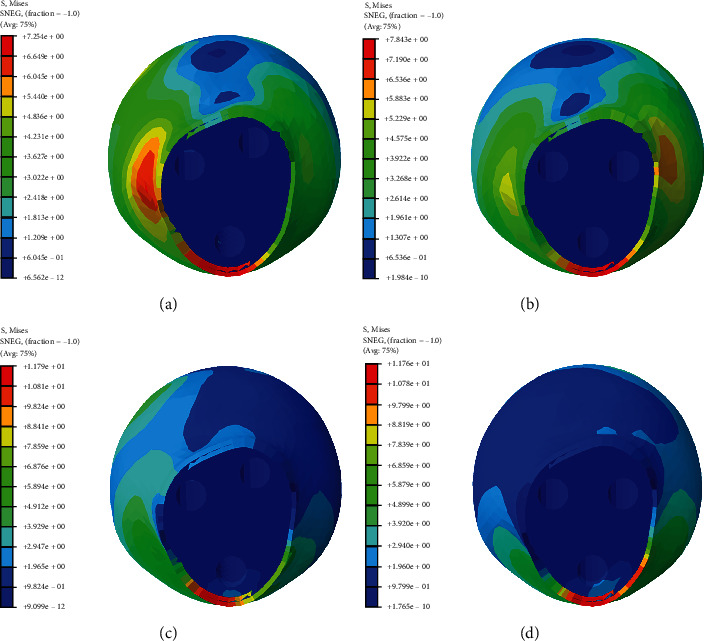
The stress nephogram of femoral head under loads of 500 N in two models. Red is the maximum stress. (a). Oblique triangle screw placement in complete femoral neck model. (b). Equilateral triangle screw placement in complete femoral neck model. (c). Oblique triangle screw placement in femoral neck fracture model. (d). Equilateral triangle screw placement in femoral neck fracture model.

**Figure 4:**
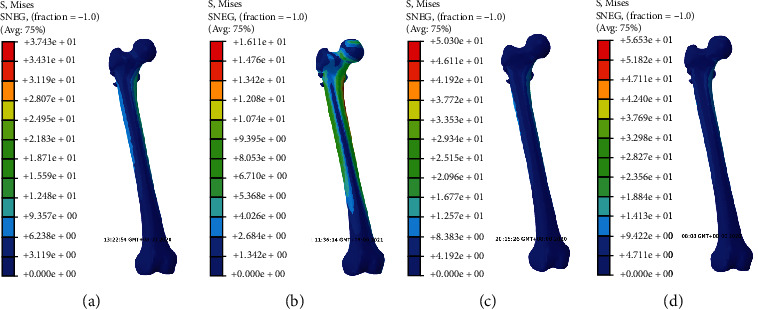
The stress nephogram of femur under loads of 500 N in two models. Red is the maximum stress. (a). Oblique triangle screw placement in complete femoral neck model. (b). Equilateral triangle screw placement in complete femoral neck model. (c). Oblique triangle screw placement in femoral neck fracture model. (d). Equilateral triangle screw placement in femoral neck fracture model.

**Figure 5:**
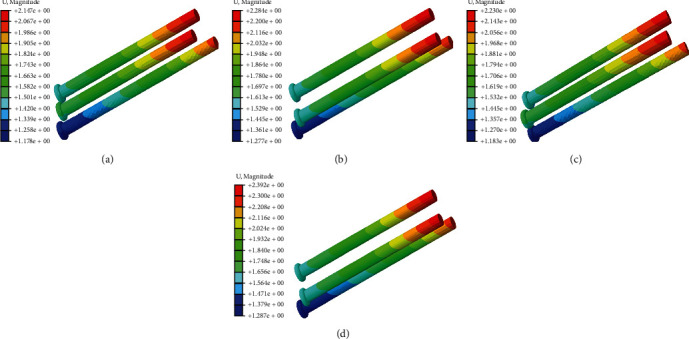
The displacement nephogram of cannulated screws under loads of 500 N in two models. Red is the maximum displacement upward and blue is the maximum displacement downward. (a). Oblique triangle screw placement in complete femoral neck model. (b). Equilateral triangle screw placement in complete femoral neck model. (c). Oblique triangle screw placement in femoral neck fracture model. (d). Equilateral triangle screw placement in femoral neck fracture model.

**Figure 6:**
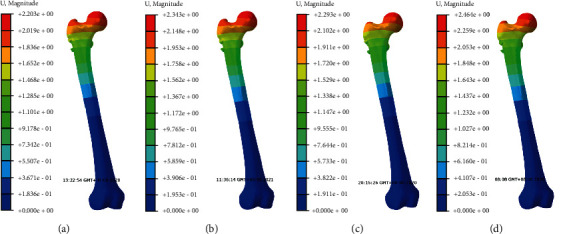
The displacement nephogram of femur under loads of 500 N in two models. Red is the maximum displacement upward and blue is the maximum displacement downward. (a). Oblique triangle screw placement in complete femoral neck model. (b). Equilateral triangle screw placement in complete femoral neck model. (c). Oblique triangle screw placement in femoral neck fracture model. (d). Equilateral triangle screw placement in femoral neck fracture model.

The stress nephogram of cannulated screws, femoral head and femur in two models were shown in Figures 2–4, respectively. The displacement nephogram of cannulated screws and femur in two models were shown in Figures 5 and 6,respectively.

### 3.2. Maximum Von Mises Stress of Cannulated Screws

**Figure 7:**
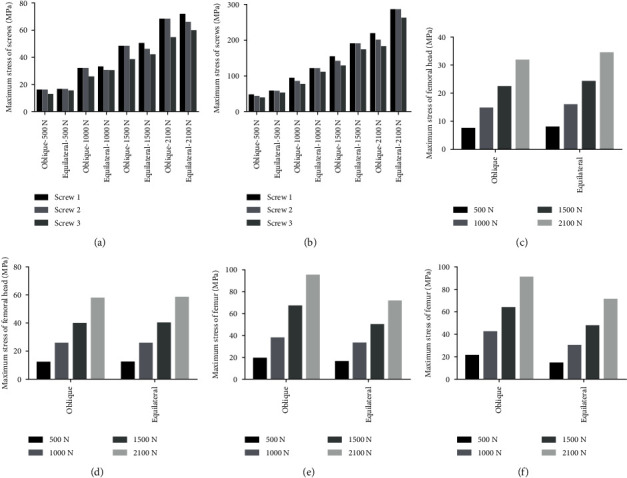
Maximum stress of cannulated screws in (a) complete femoral neck model and (b) femoral neck fracture model. Maximum stress of femoral head in (c) complete femoral neck model and (d) femoral neck fracture model. Maximum stress of femur in (e) complete femoral neck model and (f) femoral neck fracture model.

In complete femoral neck model, the maximum Von Mises stress of the three cannulated screws of equilateral triangle screw placement was slightly larger than that of oblique triangle screw placement, and this difference was more obvious in femoral neck fracture model (Figures 2, 7(a), and 7(b)).

### 3.3. Maximum Von Mises Stress of Femoral Head

The stress distribution of the femoral head was generally downward, which was more uniform in the complete femoral neck model and more concentrated in the lower side in the femoral neck fracture model. The peak stress was at the fracture line (Figure 3). In femoral neck fracture model, there was no difference between the stress distribution of the femoral head and the Von Mises peak stress in the case of equilateral triangle screw placement and oblique triangle screw placement (Figures 7(c) and 7(d)).

### 3.4. Maximum Von Mises Stress of Femur

The stress peaks of all models were concentrated at the contact site between the inner side of the femur and internal fixation, and near the proximal medial femur and the lesser trochanter. In all models, the overall maximum Von Mises stress of femur was greater under the oblique triangle screw fixation than that under equilateral triangle screw fixation (Figures 4, 7(e), and 7(f)).

### 3.5. Maximum Axial Displacement of Cannulated Screws

**Figure 8:**
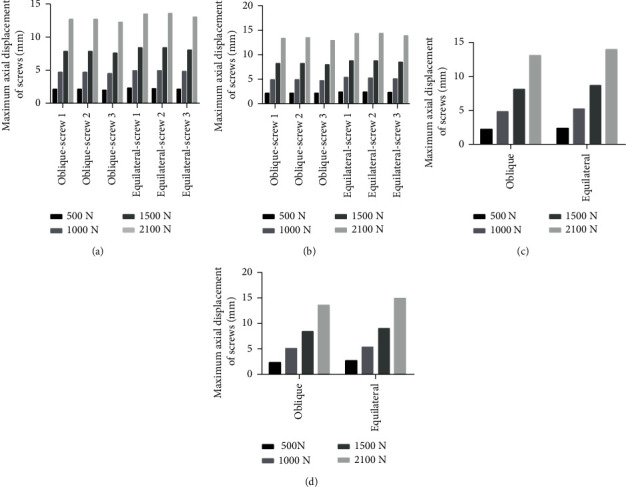
Maximum axial displacement of cannulated screws in (a) complete femoral neck model and (b) femoral neck fracture model. Maximum stress of femur in (c) complete femoral neck model and (d) femoral neck fracture model.

In all models, the maximum screw displacement of was at the tip of the screw, which increased with the increase of loads. The screw displacement in femoral neck fracture model was slightly larger than that of complete femoral neck model. Under same loads, the maximum displacement of each screw with oblique triangle screw placement was lower than that of equilateral triangle screw placement in complete femoral neck and femoral neck fracture model (Figures 5, 8(a), and 8(b)).

### 3.6. Maximum Axial Displacement of Femur

In both models, the overall displacement distribution of the femur was similar, with the maximum displacement at the top of the femoral head and increasing with the increase of applied loads. The overall displacement in femoral neck fracture model was greater than that of complete femoral neck model. Under same loads, the overall maximum displacement of femur in oblique triangle screw placement was less than that of equilateral triangle screw placement in the two models (Figures 6, 8(b), and 8(c)).

## 4. Discussion

Studies have shown that the three-dimensional shape of the femoral neck is irregular [11], not cylindrical or triangular, but is triangular prism in the proximal end and quadrangular prism in the distal end. We found that in the clinical application of cannulated screw treatment of femoral neck fractures, the posterior cannulated screws are penetrated from the back and upper slope of the femoral neck however cannot be observed by intraoperative fluoroscopy. The configuration of the cannulated screws should conform to the normal physiological shape of the femoral neck to obtain better stability. Therefore, we believe oblique triangle placement of cannulated screws, not the inverted triangle, should be used for femoral neck fractures, in which the posterior upper screw was lower than the anterior upper screw. It is expected that a better result may be obtained by placing three cannulated screws in the posterior oblique triangle location formed by the longitudinal axis of the upper femur and the long axis of the ellipse [12].

The maximum Von Mises stress of the three cannulated screws of equilateral triangle screw placement was slightly larger than that of oblique triangle screw placement in both models, showing that the model with oblique triangle placement is less stressed than equilateral triangle screw placement under fracture internal fixation. Compared with the complete model, the maximum Von Mises stress of each screw is higher in the fracture model, and the maximum stress was concentrated at the fracture end. The stress concentration of the upper two screws was more obvious, indicating the internal fixation could play a better supporting role and the stress of femur was reduced. The overall maximum displacement of oblique triangle screw placement was less than that of equilateral triangle screw placement in the two models under same loads, which showed better stability of oblique triangle fixation.

Our research showed that for people in high-altitude areas, the three screws should be implanted in an oblique triangle configuration other than equilateral triangle screw fixation. For oblique triangle configuration, the anterior upper screw should be at the junction of the narrowest plane, the posterior upper screw is lower than the anterior upper screw, and close to the posterior cortex. The best angle between the two points and the horizontal plane is about 20°. The lowest screw is implanted along the lowest cortex. The angles of the formed triangle are 47.63°, 75.49°, and 56.88°, respectively. In this case, the three screws can achieve the best stabilization effect.

Previous study showed [13] that in the morphological measurement of the proximal femur of Japanese, the depth of the femoral head on the coronal plane of the femur is about 25 mm at both the upper and lower screw positions. This is important because if the depth is shorter than the hip thread, the fracture line will be within the thread and therefore the compression force will not work between the fragments which ended with failed fixation. In clinical cases, the screw tip is usually placed about 5 mm from the subchondral bone. Therefore, the effective depth of the thread will be 20 mm. Therefore, the recommended thread length should be 20 mm or less to prevent the thread from passing through the fracture line.

There were some limitations in this study. First, our research object is population living in the high-altitude area of Qinghai. Due to the different geographical and ethnic groups in different high-altitude areas, we are not sure whether our suggestions can be uniformly applied in all patients with femoral neck fractures at high altitudes. This remain to be further determined. Second, due to the limitations of the three-dimensional finite element technology, we cannot account the critically important anatomical variables of the internal anatomy (bone architecture) which is unique for the proximal femur and the model assumes the cancellous bone of the femoral neck as the homogenous tissue, but this is not the case in reality.

## 5. Conclusion

For people in high-altitude areas, the three screws should be implanted in an oblique triangle configuration other than equilateral triangle screw fixation in the clinical application of cannulated screw treatment of femoral neck fractures.

## Figures and Tables

**Table 1:** Materials and properties of finite element models in this study.

Structure	Modulus of elasticity (GPA)	Poisson's ratio
Ti-6AL-4 V	105	0.35
Cortical bone	16.80	0.30
Cancellous bone	0.84	0.20

**Table 2:** Results of measurement data.

	Min	Max	Range	Mean	SD
∠a	15.43	24.669	9.24	20.26	2.33
∠A	38.33	69.405	31.08	47.63	4.43
∠B	63.73	84.599	20.87	75.49	4.36
∠C	43.23	69.824	26.60	56.88	5.30

## Data Availability

The authors confirm that the data supporting the findings of this study are available within the article.

## Abbreviations

CT: Computed tomography.
